# Fast and easy method to culture and obtain large populations of male nematodes

**DOI:** 10.1016/j.mex.2023.102293

**Published:** 2023-07-20

**Authors:** Justine Cailloce, Fanny Husson, Aniela Zablocki, Vincent Galy, Jorge Merlet

**Affiliations:** Sorbonne Université, CNRS, Institut de Biologie Paris Seine (IBPS), Developmental Biology Laboratory, UMR 7622, Paris, France

**Keywords:** Purification, *C. elegans*, Reproduction, Filtering, Culture, Nematode males purification by filtering

## Abstract

*Caenorhabditis elegans* is a model system widely used in fundamental research. Even though, nematodes are easy to maintain in the laboratory, obtaining large populations of worms require a lot of work and is time consuming. Furthermore, because *C. elegans* are mainly hermaphrodite it is even more complicated to obtain large amounts of males which make high-throughput experiments using *C. elegans* males very challenging.

In order to overcome these limitations, we developed affordable and rapid methods to:

(1) grow large synchronous worm populations

(2) easily obtain large amounts of males

We developed a culture method on plates to grow big synchronized worm populations with the standard incubators used on all worm labs. We also established an easy filtration method allowing to obtain large male populations in an hour. After filtering, the worm population contains more than 90% of adult males and no adult hermaphrodites since all the contaminants are larva and embryos.

The culture and the filtering methods we developed are easy to implement and require a very limited investment in equipment and consumables beside the standard one present in worm labs. In addition, this filtering method could be applied to nematode's species similar in size to *C. elegans*.

## Graphical abstract

**Figure:**
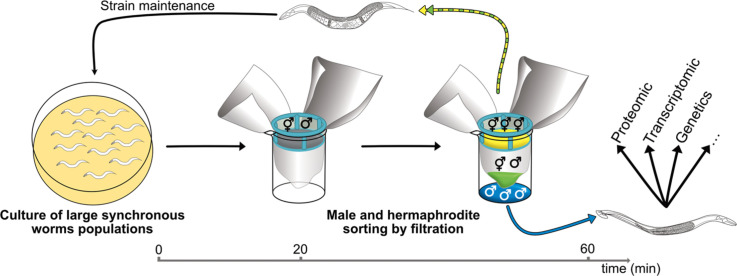

Specifications table

**Table:** 

Subject area:	Biochemistry, Genetics and Molecular Biology
More specific subject area:	Reproduction, male high throughput analysis
Name of your method:	Nematode males purification by filtering
Name and reference of original method:	NA
Resource availability:	NA

## Method details

*C. elegans* are mainly hermaphrodite with some rare males in the population [1]. The rate of males can be increased with some genetic mutation a.k.a *him* mutants [2]. The low fraction of males in *C. elegans* populations makes very challenging to obtain large amounts of males. Furthermore, liquid culture is commonly use to obtain large populations of worms however, maintaining worms in liquid culture is not trivial, time consuming and requires specific and dedicated equipment (at least one shaking incubator with a cooling system to keep the culture temperature between 15° and 25 °C). Importantly, worm physiology is quite different in liquid culture from those on solid media which could be a limitation for some experiments. Another method to purify males using one filter had been very briefly described [3]. This method was not very easy to handle, poorly efficient in our hands and it used 'egg plates' to grow large populations of worms on solid media, which are very awkward to work with. Another study to recover large populations of male worms employed inducible degradation of a dosage compensation protein to selectively kill hermaphrodites [4]. However, this method requires the insertion of a transgene on your favourite *C. elegans* strain and the use of a drug which are not always possible or might be impossible if you are working on another nematode specie. Alternatively, males can be sorted using a worm sorter, an equipment not available in a classical worm laboratory and it requires also to introduce a male specific fluorescent transgene on your strain which could be an issue as mentioned above. In order to solve these problems, we developed a convenient and standardized culture method on solid media for large populations of worms coupled to a rapid filtering protocol to easily obtain large populations with more than 90% of males without the need for specific nor expensive equipment.

## Plate preparation

(1) Worms are cultivated on 145 mm diameter NGM (Nematode Growth Media) agar plates enriched in peptone (NaCl 3 g/L; agar 25 g/L; cholesterol 5 mg/L; CaCl_2_ 1 mM; MgSO_4_ 1 mM; buffer K_2_HPO_4_/KH_2_PO_4_ 25 mM (pH=6) and peptone 20 g/L).
(2) Plates are seeded with 1 mL of concentrated bacteria culture of NA22 *E. coli* (NA22 (25x)). Seeded plates are incubated at RT for at least 48 h before use.

NOTE: NA22 are cultivated in 2xYT liquid media (NaCl 5 g/L; tryptone 16 g/L; yeast extract 10 g/L) ON at 37 °C, 200 rpm and 25-times concentrated to obtain the NA22 (25x) suspension.

NOTE: To recover the optimal amount of NA22 a maximum of 500 mL of culture must be grown in a 2 L flask culture.

NOTE: Spread the NA22 bacteria evenly over the agar surface. Incubate at RT for 48 h at least is crucial to avoid the worms to starve during the culture.

NOTE: Other *E. coli* strains than NA22 can be used but the quantity needed will have to be carefully adjusted. NA22 bacteria are convenient to use to grow such large populations of worms because they are prototroph. With OP50 a much larger volume of bacteria culture will be required in order to obtain enough food to allow the worms to reach adulthood without starving.

### Culture of large synchronous populations of *C. elegans* on plates

(1) Add 140 000 L1 larvae (L1) per 145 mm diameter seeded plate.

NOTE: To obtain L1, collect from 10 plates with asynchronous worms maintained under standard worm laboratory conditions [5] and extract the embryos by controlled lysis using a bleaching solution (Bleach 0.5%, NaOH 0.7 M). Let the embryos hatch ON in M9 buffer (Na_2_HPO_4_,12H_2_0 17 g/L; KH_2_PO_4_ 3 g/L; NaCl 5 g/L; MgSO_4_ 1 mM) to obtain L1 synchronous population. Count the L1 and plate around 8 000 worms on 90 mm diameter NGM agar plates seeded with 1 mL NA22 (25x). Let them grow until they reach adulthood. Bleach five to ten 90 mm plates to obtain 140 000 L1.

NOTE: In order to increase the initial number of males we used CU607 strain (*smIs23* [pkd-2::gfp] II*; him-5(e1490)* V) [6]. The *him-5(e1490)* allele increases the frequency of males to 30% [2]. We obtained similar results with different strains containing the *him-5(e1490)* allele or the *him-8(e1489)* allele.

NOTE: CU607 contains also *smIs23* transgene which allows a male specific GFP expression in neurons. This marker is not required for this method but can be used after the filtration to sort GFP males on a worm sorter (Union Biometrica) and reach 100% male purity.

(2) Synchronized worms are grown for 3 days at 20 °C or 5 days at 15 °C until they reach adulthood.

NOTE: Most worms strain we tested reached adulthood more synchronously when grown at 15 °C. The temperature to grow the worms must be adjusted for each strain in order to obtain the most synchronous population.

(3) Wash worms from the plate with 10 mL M9 buffer and transfer them into a 15 mL centrifuge tube.
(4) Let them sediment completely for 3–5 min at room temperature (RT).

NOTE: Avoid centrifugation to better remove bacteria embryos and small larvae (L1, L2).

(5) Remove the supernatant containing the bacteria, embryos and small larvae (L1, L2).

NOTE: Repeat steps 4) and 5) two or three times until the supernatant is completely clear. Resuspend the worms by inverting the tube 2/3 times.

(6) Add 10 mL of bleaching solution (Bleach 0.5%, NaOH 0.7 M) for 7 min with agitation.

NOTE: Worms pellet should not exceed 500 µL, otherwise split the pellet in several tubes.

(7) Centrifuge 1 min at 100 g.

NOTE: Work under sterile condition from this step.

(8) Remove the supernatant, add 10 mL of sterile M9 buffer, resuspend worms and centrifuge 1 min at 100 g. Repeat this step 3 times.
(9) After the final wash remove the supernatant, resuspend embryos in 13 mL of M9 buffer and transfer them to a 100 mL Erlenmeyer.
(10) Let the embryos hatch ON under agitation (120 rpm) to obtain L1 synchronous population.
(11) Count the number of L1 larvae.

NOTE: To have synchronous worm populations do not keep L1 in M9 buffer more than 24 h.

## Male and hermaphrodite separation

(1) Add 140 000 L1 on a 145 mm diameter seeded plate and let them grow until adulthood.

NOTE: Stop the culture when all the worms are adults and you don't see L4 anymore. L4 and males can't be discriminated by the filters.

(2) Recover the worms from the plate in 15 mL of M9 buffer in a 15 mL centrifuge tube.

NOTE: Wash the plate twice. Recover both washes in the same 15 mL centrifuge tube, with a final volume of 15 mL. If necessary, wash the plate again until there are no more worms on the plate.

(3) Let the worms sediment.

NOTE: Do not centrifuge to avoid bacteria sedimentation and improve bacteria removal.

(4) Remove the supernatant containing the bacteria and embryos and repeat steps 3) and 4) until the supernatant is fully clear.

NOTE: Resuspend the worms in 10 mL M9 buffer by inverting the tube 2/3 times between each wash.

**Fig. 1:**
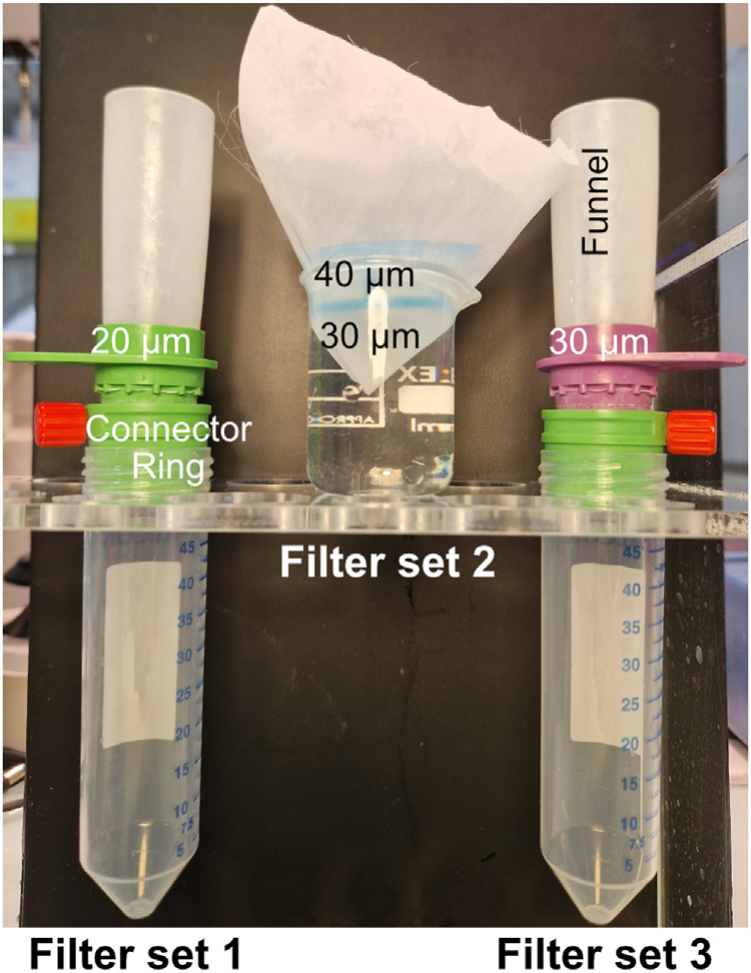
**Filtration devices.** A photography of the three devices (filters set 1, 2 and 3) used is shown. Filters set 1 and 3 are both used for washes to remove bacteria, embryos and L1-L3 larvae. A strainer (20 µm or 30 µm) is attached to the connector ring which is attached to a 50 mL tube. A funnel is inserted in the strainer. The connector ring allows to control the flow speed. The Filter set 2 is used to separate hermaphrodites and males. On a 25 mL beaker we assembled a 30 µm membrane filter (12 cm x 12 cm) and a 40 µm Cell Strainer. The beaker is filled with M9 only once the filters are mounted.

NOTE: Always add a small volume of M9 buffer to the funnel before adding the suspended worms.

(5) Add 10 mL of M9 buffer to resuspend worms.
(6) Prepare 3 types of filters (Fig. 1):
  - Filter set 1: A 50 mL centrifuge tube with a 20 µm strainer filter (#43–50020–03, Pluriselect), a connector ring (#41–50000–03, Pluriselect) and a funnel (#42–50000, Pluriselect). Prepare the filter by passing 5 mL of M9 buffer, close the connector ring.
  - Filter set 2: A 25 mL beaker with a 30 µm membrane filter (12 cm x 12 cm, #11735488 Fischer Scientific) over a 40 µm Cell Strainer (#431750, Corning).

NOTE: Fill the beaker with M9 buffer but leave enough volume to add the worms.

NOTE: Be careful not to leave any air bubbles under the 40 µm Cell strainer.

- Filter set 3: A 50 mL centrifuge tube with a 30 µm strainer filter (#43–50030–03, Pluriselect), a connector ring (Pluriselect) and a funnel (Pluriselect). Prepare the filter by passing 5 mL of M9 buffer, close the connector ring.

NOTE: This step can be done earlier in the protocol.

(7) Add 5 mL of M9 buffer to the filter set 1 (with connector ring closed).

(8) Add the 10 mL of worms resuspended in M9 buffer.

NOTE: Load worms from a maximum of one plate (140 000 worms) / filter set to avoid the overload of the filters.

(9) Open the connector ring to let the buffer gently pass through the strainer filter 1. Control the flow manually by closing or opening the connector ring. Let pass all the buffer until the worms are left in a few µL buffer.
(10) Close the connector ring and fill the funnel with M9 buffer. The worms will resuspend. Open the connector ring to let the buffer gently pass. Repeat the step until you washed with 40 mL of M9.
(11) Let the worms in M9 buffer (< 1 mL). Gently remove the funnel.

NOTE: This first filtration step removes embryos, larvae, and persistent bacteria. This filtration can be multiplied to limit the presence of contaminating embryos at the end of the protocol.

(12) Recover the worms from the strainer filter with a glass Pasteur pipette and gently transfer them to the filter set 2.
(13) Rinse the filter set 1 with 500 µL of M9 buffer to recover all the worms.

NOTE: Be careful not to crush the worms against the strainer filter when pipetting them with the Pasteur pipette.

(14) Allow the worms to pass through the filters for 40 min at RT.
(15) Collect the worms (population #1) on the 40 µm Cell Strainer filter with a new glass Pasteur pipette and transfer them in a new 15 mL centrifuge tube.

NOTE: The recovered population #1 is composed almost exclusively of hermaphrodites and can be used for strain maintenance.

(16) Recover the worms present on the 30 µm membrane filter (population #2) with a new glass Pasteur pipette and place them in a new 15 mL centrifuge tube.

NOTE: Be careful not to open or move the membrane filter to avoid to contaminate the male population which is at the bottom of the beaker. The recovered population #2 is composed of hermaphrodites and males and can also be used for strain maintenance.

(17) Remove gently the membrane filter and remove almost all the buffer from the beaker.

NOTE: The males should be on the bottom of the beaker. You can remove as much buffer as you can to decrease embryo and larvae potential contamination.

(18) Collect worms (population #3) from the bottom of the beaker with a new glass Pasteur pipette and place them in a new 15 mL centrifuge tube. Rinse the bottom of the beaker with 1 mL of M9 buffer to collect remaining worms.

NOTE: The recovered population #3 is composed almost exclusively of males.

(19) Resuspend the worms in 6 mL of M9 buffer.
(20) Add 5 mL of M9 buffer to filter set 3 (with the ring connector closed) and add population #3 worms. Wash the 15 mL centrifuge tube with 5 mL of M9 buffer and transfer it to the funnel.
(21) Open the connector ring to let buffer gently pass through the strainer filter set 3. Control the flow manually by closing or opening the connector ring. Let pass all the buffer until the worms are left in a few µL buffer.
(22) Close the connector ring and fill the funnel with M9 buffer. Open the connector to let the buffer gently pass. Repeat the step until you washed population #3 with 40 mL M9. Gently remove the funnel.

NOTE: This filtration step removes remaining embryos and larvae. It can be repeated in order to limit the presence of contaminating embryos at the end of the protocol.

(23) Recover the worms from the filter set 3 with a new glass Pasteur pipette and transfer them to a new 15 mL centrifuge tube. Rinse the filter set 3 with 500 µL of M9 buffer to recover all the remaining worms.
(24) The worms recovered are your final male worm population. They can be resuspended in the desired volume of M9 buffer and used for experiments.

## Method validation

**Fig. 2:**
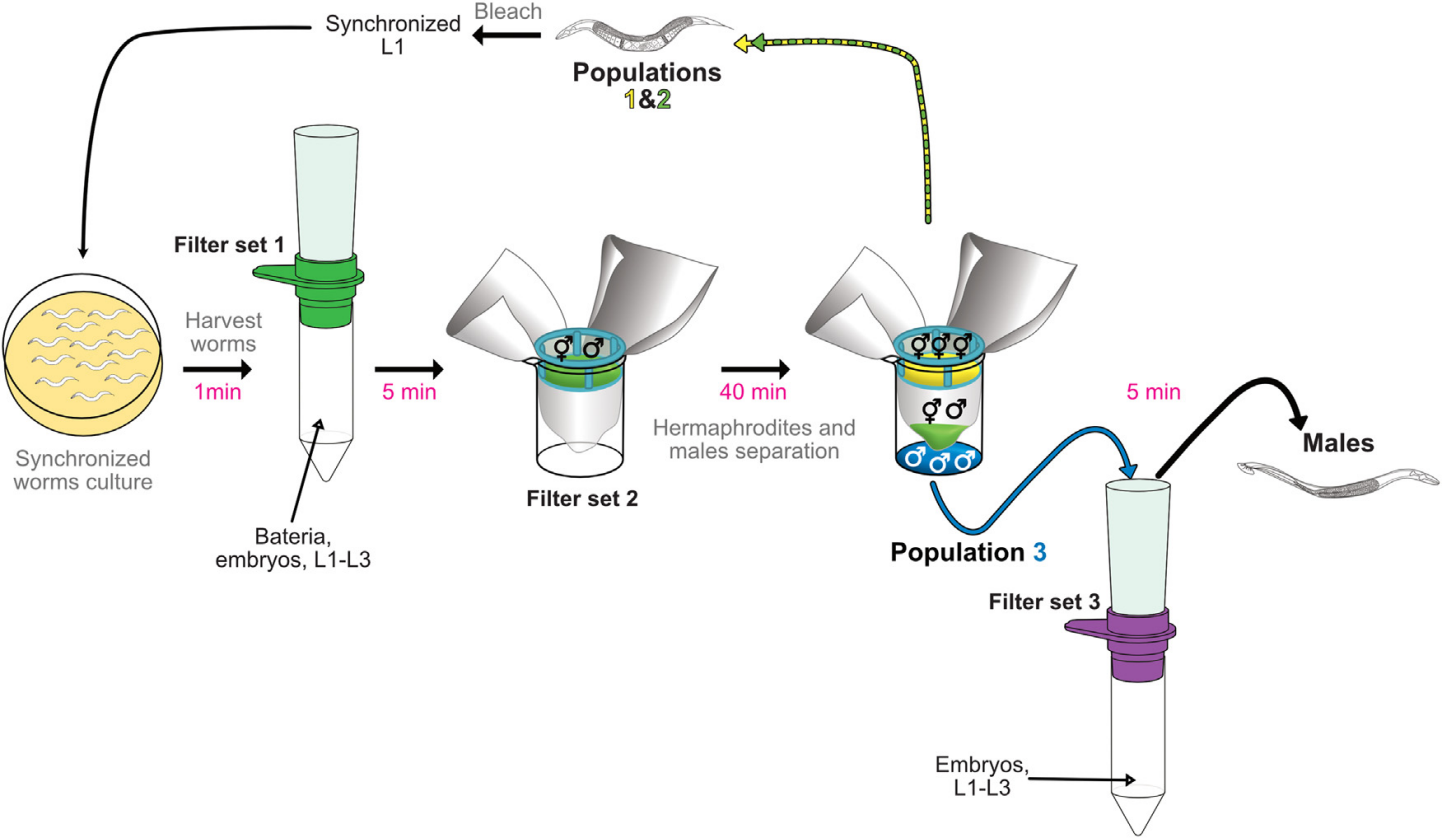
**Scheme of the male filtration protocol.** Synchronized adults’ worms are collected, washed twice on M9 (not shown). Then worms (grey) are transferred on filter set 1 (contains a 20 µm strainer) and washed with 40 mL M9 to remove the remaining bacteria, and majority of embryos and L1-L3 larvae. The funnel is removed and worms retained (grey) on the strainer are transferred to filter set 2. After 40 min incubation at room temperature three populations of worms are recovered: *top:* adults’ hermaphrodites (yellow, Population #1) retained by the 40 µm Cell Strainer, *middle*: a mixed population with males and adults’ hermaphrodites (green, Population #2) retained by the 30 µm membrane filter and *bottom:* the males (blue, Population #3) that can pass both filters. The population #3 is transferred on filter set 3 (contains a 30 µm strainer) and washed with 40 mL of M9 to remove remaining L1-L3 larvae and embryos. The funnel is removed and worms retained (black) on the strainer are the final male population. The populations #1 and #2 can be used for strain maintenance.

**Fig. 3:**
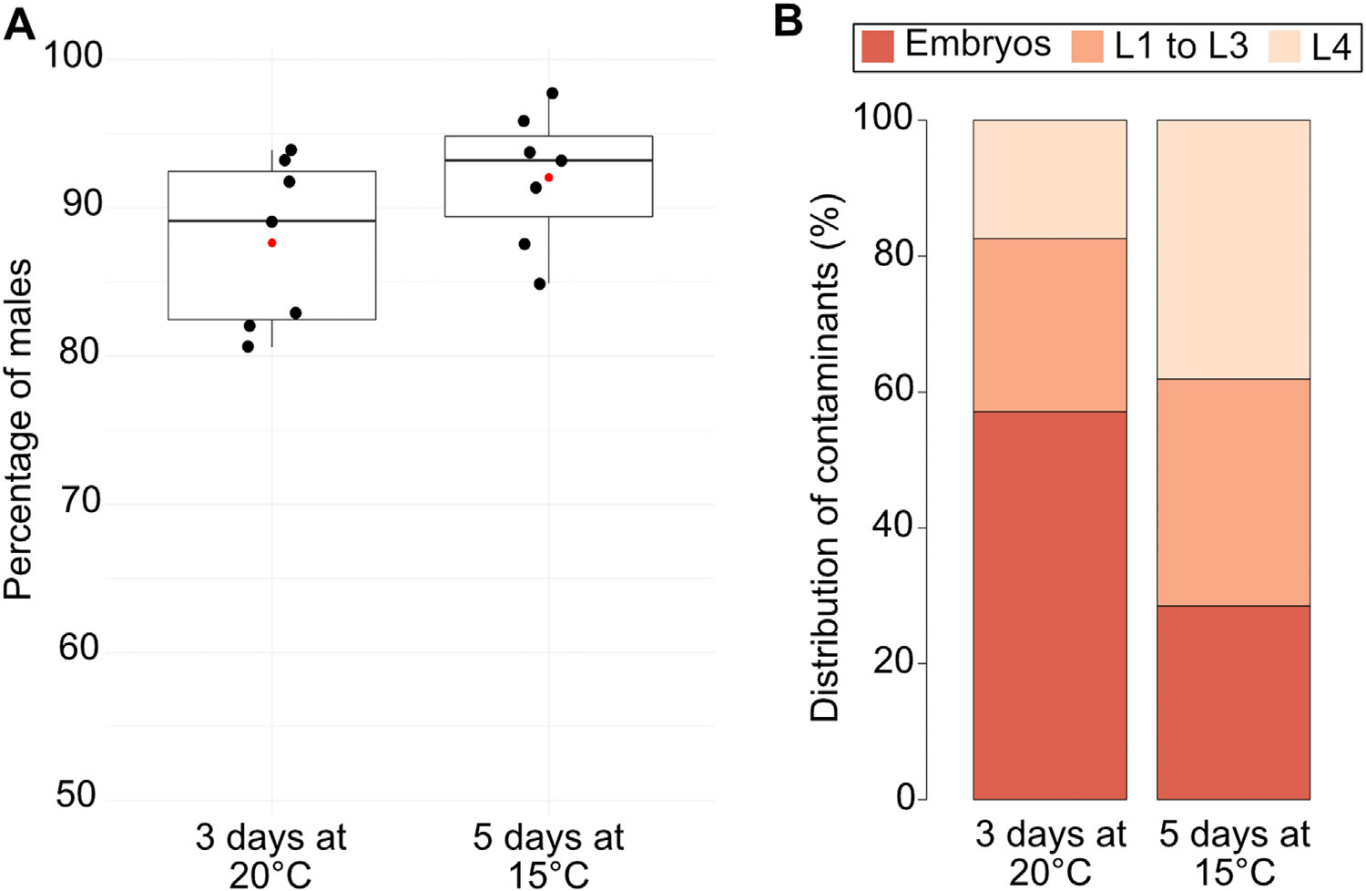
**Efficiency of the male sorting method.** Synchronized worms were grown 3 days at 20 °C or 5 days at 15 °C, and filtered. **(A)***Percentage of males*. After the filtration protocol the mean of males’ purity (red dot) obtained is 87.63% +/- 5.67 (*n* = 7) and 92.04% +/- 4.53 (*n* = 7) respectively. The initial percentage of males was 52.1% +/- 9.98 at 15 °C and 53.4% +/- 6.57 at 20 °C. **(B)***Proportion of contaminants.* The percentage of each contaminant (embryo, L1-L3 larvae and L4 larvae) after filtration (*n* = 7) was assessed.

We developed a rapid, easy method (Fig. 2) to obtain large populations with more than 90% of males (Fig. 3A). The purity in males of the population obtained with this method was high without reaching hundred percent of males. We observed three categories of contaminants, the L4 larvae, L1-L3 larvae and embryos. Indeed, it was not possible to discriminate males and L4 with filters because their diameter was very similar 49.1 +/- 2.75 µm (*n* = 10) and 49.1 +/- 3.03 µm (*n* = 7) respectively, whereas hermaphrodites had a diameter of 76.7 +/- 2.73 µm (*n* = 5). It was therefore critical to sort a very synchronous population containing only adults and we obtained better results by growing the worms at 15 °C and filter the population 30 h after they reached the L4 stage. These parameters must be adjusted to each strain. In addition to a better synchronization of the population, the total number of contaminants per 1 000 males recovered was also reduced at 15 °C compared to 20 °C, 84.5 +/- 40.67 (*n* = 7) and 131.5 +/- 75.03 (*n* = 7) contaminants respectively. As a result, the percentages of purity when the worms were grown at 15 °C and 20 °C were respectively 92.04% +/- 4.53 (*n* = 7) and 87.63% +/- 5.67 (*n* = 7) (Fig. 3A). At 15 °C, we were able to recover 52% (*n* = 7) of the males present in the initial population. We also observed that filtering the population after a longer incubation time was also improving the purity of the male population after sorting but led on recovering older males, which could be a problem for some experiments. Furthermore, extended cultures also increased the amount of L1 to L3 larvae in the initial population on plates and required therefore more washes on filter set 1 and 3. Importantly, embryos and L1 to L3 larvae were the most important kind of contaminants we found (Fig. 3B). Embryos were certainly mostly laid during the 40 min incubation on filter set 2. Once laid they sedimented at the bottom together with the males. Therefore, if needed, the purity can be increased by adding more washes on filter set 3.

We developed this method on large worm populations but the same method was also used for smaller populations with a similar efficiency since we obtained a male purity of 93% +/- 8.15 (*n* = 6) with a starting population of 5 000 worms. We also tested our filtering method to recover males from population with fewer males. Indeed, we noticed that when worms containing the *him-5(1490)* allele were maintained in large populations the percentage of males increased over several generations time to 50%. Therefore, we artificially decreased the number of males in the initial population to 20% and 5% by adding hermaphrodites for populations of around 120 000 animals. After filtration, we enriched respectively 3 times (*n* = 4) and 9 times (*n* = 2) the number of males from starting populations. Therefore, this method is efficient to obtain large and almost pure populations of males as well as to increase the fraction of males within populations where they are initially largely outnumbered.

Ethics statements

## CRediT authorship contribution statement

**Justine Cailloce:** Conceptualization, Methodology, Investigation, Formal analysis, Visualization, Writing – original draft, Writing – review & editing. **Fanny Husson:** Investigation, Validation. **Aniela Zablocki:** Conceptualization, Methodology, Investigation. **Vincent Galy:** Conceptualization, Writing – review & editing, Visualization, Funding acquisition. **Jorge Merlet:** Conceptualization, Methodology, Investigation, Formal analysis, Writing – original draft, Writing – review & editing, Visualization, Supervision.

## Declaration of Competing Interest

The authors declare that they have no known competing financial interests or personal relationships that could have appeared to influence the work reported in this paper.

## Data Availability

No data was used for the research described in the article.
